# A Case of the Diabetes Mellitus, Which Terminated in a Complete, and, as Far as Can Be Judged from Apparent Circumstances, a Permanent Cure, by Medicines, Abstracting Oxygen from the System, and a Diet Consisting Totally of Animal Matter

**Published:** 1799-05

**Authors:** R. Redfearn

**Affiliations:** Lynn Regis, Norfolk


### *A Case of the Diabetes Mellitus, which terminated in a complete, and, as far as can be judged from apparent Circumstances, a permanent Cure, by Medicines, abstracting Oxygen from the System, and a Diet consisting totally of Animal Matter:—By* R. Redfearn, *M. D. of Lynn Regis, Norfolk.*

AFTER perusing the first edition of Dr. Rollo's publication on the Diabetes Mellitus, in two volumes octavo, printed by Dilly, Mr. Spurging, a respectable farmer at Docking in this county, came to consult me, about the beginning of the year 1798, in a case of this disorder, which he had laboured under, for near two years before that time. Previous to his application for advice to me, he had consulted Dr. M. of this town, and also Dr. L. of Norwich, both of them gentlemen of eminence in their profession, without experiencing the least alleviation of his complaint. Upon Mr. Spurging's applying to me, it instantly occurred to my mind, that the nature of his disorder would afford a fair opportunity of trying the method recommended by Dr. Rollo, for treating this obstinate and troublesome disease. I therefore immediately suggested to him the propriety, and the absolute necessity, of abstaining rigidly from all fermented liquors and vegetables, with every thing else that could impart oxygen to the system by the primæ viæ; and at the same time ordered that his diet should consist principally of fat beef, pork, and such aliments as were of a gross or unctuous quality, and most likely to produce hydrogen in the greatest abundance. My patient, dejected by a long and painful affliction, and extremely anxious to recover, complied with every prescription, whether in a dietetic or medicinal point of view, with the strictest care and attention; declaring that the most nauseous medicines could not shake his resolution from persevering in any course of physic, or regimen, which might be adopted towards his recovery. Deeply affected by his sufferings, and desirous of contributing to his relief, I began with prescribing to him, when thirsty, to drink very weak brandy and water, with ten or twelve drops of the hepatised ammonia, a bottle of which he was furnished with, prepared as directed by Mr. Cruickshank, in Dr. Rollo's publication. This he was desired to augment gradually, as his stomach became habituated to its stimulus; and the following medicines were administered:—

| | | |
|---|---|---|
| ℞ | Pulveris cinchonæ rub. — — | unc. ii. |
| | Aluminis usti — — — | drachm. iii. |
| | Kali præpariti — — — | drachm. ij. |
| | Petrolei sulphurati — — — | q. s. |

Misce,

Misce, fiat elect. de quo capiat nucis moschatæ molem ter in die, horis duabus ante et post prandium; et hora septimâ vespertinâ superbibat cyathum misturæ sequentis:—

| | | | | | |
|---|---|---|---|---|---|
| R/ | Aquæ calcis | — | — | — | unc. xvi. |
| | Kali sulphurati | — | — | — | drachm. iii. |

Fiat mistura.

| | | | | |
|---|---|---|---|---|
| R/ | Natron præparati lenè calcinati | | — | drachm. i. |
| | Saponis albi Hispani | — | — | scrup. iv. |
| | Mucilaginis gummi Arabici | | — | q. s. |

Misce, fiant pilulæ triginta, quarum capiat quatuor singulâ nocte.

It would be altogether superfluous, and perhaps impossible, to enumerate the various diagnostics concomitant on this disease, in different subjects. The most prominent symptoms, that attended my patient, I shall, however, briefly recount, viz. an intolerable thirst; parched skin; tongue whitish, and moist on its exterior surface, but reddish on the external edges; saliva white, frothy, and extremely viscid, so as to render exspuition very difficult; the profluvium urinæ limpid, and of a saccharine quality, and voided upon an average to the quantity of six or seven quarts in the course of twenty-four hours. The wasting of the muscular parts had been gradual from the very first commencement of the disease; and from a corpulent habit he was reduced to a state bordering upon emaciation.

After this patient had persevered in the above medicines and regimen during a fortnight only, he found his thirst by no means so excessive. The quantity of his urine was considerably diminished, and became also of a quality more urinous, and less sweet. His amendment continued to be progressive, without feeling any interruption, either from natural or adventitious causes; and he was completely free from every symptom of the disease in less than three months after the medicines were first administered. He never once deviated from the regimen prescribed; nor omitted taking his medicines regularly, although they were extremely unpleasant. The hepatised ammonia was increased to thirty or forty drops at a dose, without his experiencing any disagreeable effects from it, and he took of this article alone, nearly ten ounces.

My patient has now continued perfectly well for more than eight months, nor has he taken any medicines since, except half a pint of SCHWEPPE's double acidulated Soda water, occasionally prescribed, with a little brandy, by way of beverage: and although he has indulged several times in fermented liquor during that period, no relapse has ensued from such indulgence, gence, and to my enquiries, very lately, he declared, that he never enjoyed a better ftate of health, than he does at this moment.

The kali fulphuratum, or fulphuret of pot-afh, fhould be carefully infpected by the faculty, when adminiftered as a medicine; becaufe, when recently prepared, it is extremely cauftic by the difengagement of the carbonic acid from the pot-afh, during the procefs of preparing it. In this ftate, more moderate dofes fhould be made ufe of; but when it has regained this aëriform, elaftic fluid, (the deprivation of which conftituted its caufticity) as it frequently does in the difpenfaries, by an expofure to the atmofpheric air, or for want of proper caution in being fecured from its contact: whenever this happens, I have always found its efficacy of little avail, either in the diabetes, or in the removal, or prevention of ptyalifm excited by mercury.

*Lynn, April 5th*, 1799.

